# Boosted Influenza-Specific T Cell Responses after H5N1 Pandemic Live Attenuated Influenza Virus Vaccination

**DOI:** 10.3389/fimmu.2015.00287

**Published:** 2015-06-02

**Authors:** YanChun Peng, Beibei Wang, Kawsar Talaat, Ruth Karron, Timothy J. Powell, Hui Zeng, Danning Dong, Catherine J. Luke, Andrew McMichael, Kanta Subbarao, Tao Dong

**Affiliations:** ^1^MRC Human Immunology Unit, Weatherall Institute of Molecular Medicine, University of Oxford, Oxford, UK; ^2^Institute of Infectious Diseases, Beijing Ditan Hospital, Capital Medical University, Beijing, China; ^3^Center for Immunization Research, Johns Hopkins University Bloomberg School of Public Health, Baltimore, MD, USA; ^4^Laboratory of Infectious Diseases, National Institute for Allergy and Infectious Disease, National Institutes of Health, Bethesda, MD, USA

**Keywords:** influenza, H5N1, vaccine, T cells, LAIV, peptide, epitope, antigenic sin

## Abstract

**Background:**

In a phase I clinical trial, a H5N1 pandemic live attenuated influenza virus (pLAIV) VN2004 vaccine bearing avian influenza H5N1 hemagglutinin (HA) and NA genes on the A/Ann Arbor cold-adapted vaccine backbone displayed very restricted replication. We evaluated T cell responses to H5N1 pLAIV vaccination and assessed pre-existing T cell responses to determine whether they were associated with restricted replication of the H5N1 pLAIV.

**Method:**

ELISPOT assays were performed using pools of overlapping peptides spanning the entire H5N1 proteome and the HA proteins of relevant seasonal H1N1 and H3N2 viruses. We tested stored peripheral blood mononuclear cells (PBMCs) from 21 study subjects who received two doses of the H5N1 pLAIV. The PBMCs were collected 1 day before and 7 days after the first and second pLAIV vaccine doses, respectively.

**Result:**

T cell responses to conserved internal proteins M and NP were significantly boosted by vaccination (*p* = 0.036). In addition, H5N1 pLAIV appeared to preferentially stimulate and boost pre-existing seasonal influenza virus HA-specific T cell responses that showed low cross-reactivity with the H5 HA. We confirmed this observation by T cell cloning and identified a novel HA-specific epitope. However, we did not find any evidence that pre-existing T cells prevented pLAIV replication and take.

**Conclusion:**

We found that cross-reactive T cell responses could be boosted by pLAIV regardless of the induction of antibody. The impact of the “original antigenic sin” phenomenon in a subset of volunteers, with preferential expansion of seasonal influenza-specific but not H5N1-specific T cell responses merits further investigation.

## Introduction

Influenza is a global public health problem, with seasonal epidemics caused by human H1N1, H3N2, and B viruses, and sporadic disease caused by avian influenza A viruses, which can lead to severe illness in humans (1–4). Live attenuated influenza vaccines (LAIV) that contain the A/Ann Arbor cold-adapted (AA ca) backbone (5) are immunogenic and protective and are licensed for protection against seasonal influenza (6–8). We have generated and evaluated candidate live attenuated vaccines for pandemic use (pLAIVs) bearing avian influenza A hemagglutinin (HA) and neuraminidase (NA) genes on the AA ca vaccine backbone. The H5N1 pLAIV (VN2004) bearing the HA and NA genes from the A/Vietnam/1203/2004 (H5N1) virus was evaluated in a phase I clinical trial (NCT00347672) (9). The infectivity of the H5N1 pLAIV was assessed by virus isolation and rRT-PCR amplification of vaccine virus from daily nasal washes and the immunogenicity of the vaccine was assessed by serologic methods including hemagglutination inhibition (HAI) and microneutralization and ELISA assays. The replication of the vaccine virus was highly restricted and the vaccine failed to elicit robust antibody responses (9).

Although antibody responses to inactivated influenza vaccine correlate with protection, several lines of evidence show that post-vaccination antibody titers are not the sole surrogate for vaccine efficacy, especially for LAIV (10–13). Several studies demonstrate that regardless of the presence of the antibody, influenza-specific T cell responses correlate with viral clearance (14, 15). The “Cleveland Family study” showed that protection from influenza correlated with T cell responses, and cross-reactive T cell responses might contribute to the protection (16). Therefore, as suggested by Schotsaert et al., the correlation of vaccine efficacy with alternative measures of immune function such as influenza-specific T cell responses warrants further attention (17).

In this study, we evaluated the T cell responses in peripheral blood mononuclear cells (PBMCs) from the cohort of study subjects who received two doses of the H5N1 VN 2004 ca vaccine approximately 50 days apart (9). T cell responses to overlapping peptide pools spanning the entire H5N1 proteome, as well as the HA proteins of relevant seasonal influenza viruses, were evaluated before and 7 days after each vaccination. We found that T cell responses with effector phenotypes were boosted by vaccination, regardless of vaccine infectivity or the serum HAI titer elicited. The potential role of pre-existing T cell responses in restricting the replication of H5N1 pLAIV was also evaluated.

## Materials and Methods

### Study population

Nineteen healthy volunteers received two doses of the H5N1 VN 2004 pLAIV approximately 50 days apart, and two additional volunteers received only one dose (9). Blood samples were taken from each of the study subject at four time points: pre-vaccination, 7 days after the first dose of vaccine, 1 day prior to the second dose of vaccine, and 7 days after the second dose of vaccine. The study subjects were divided into two groups according to their infection status. Infection with the vaccine virus was inferred if the study subjects shed vaccine virus in culture, were RT-PCR positive after day 1, and/or demonstrated a fourfold or greater rise in serum antibody titer (9). Ethical approval was obtained from the Committee on Human Research Institutional Review Board (IRB) of the Johns Hopkins Bloomberg School of Public Health and the Institutional Biosafety Committee of Johns Hopkins University. Informed consent was obtained from all participating individuals prior to the study (9). The ClinicalTrials.gov identifier for this study is NCT00347672.

In order to understand the priming of the immune system induced by the H5N1 pLAIV, subjects who received two doses of the H5N1 VN 2004 pLAIV were contacted 4 years after receipt of the pLAIV and invited to participate in a follow-up study. Eleven subjects returned for this additional booster dose of 45 μg of the H5N1 inactivated unadjuvanted subvirion influenza vaccine (ISIV) (NCT01109329) (18). Antibody responses were measured after the boost dose, and compared with subjects who had received a non-H5N1 pLAIV or the ISIV alone (18).

### Synthetic peptides for T cell analysis

A total of 890 15- to 18-mer peptides overlapping by 10 amino acid residues and spanning the full avian influenza H5N1 proteome and seasonal influenza H3N2/H1N1 HA proteins was synthesized by Sigma-Aldrich (Haverhill, Suffolk, UK) and used in our previous study (19). The peptides were dissolved in DMSO (Sigma-Aldrich) at 40 mg/ml and diluted with RPMI 1640 (Sigma-Aldrich) to a concentration of 2 mg/ml (long-term stock, stored at −80°C) before being individually filtered and combined into different pools: H1 HA, H3 HA, H5 HA, M, and NP (40–90 peptides/pool).

### *Ex vivo* IFNγ ELISPOT assay

Cryopreserved PBMCs were thawed in a 37°C water bath and re-suspended in RPMI 1640 supplemented with 2% v/v heat-inactivated fetal calf serum (FCS, Sigma-Aldrich), 2 mM l-glutamine (Sigma-Aldrich), 1% v/v (100 U/ml) penicillin streptomycin (Sigma-Aldrich) (R2 medium), and 60 μg/ml DNase solution (Type IV, Sigma-Aldrich) for 15 min at 37°C. Cells were washed and re-suspended in R10 medium (RPMI1640, 10% FCS, 2 mM l-glutamine, and 1% PenStrep) and rested overnight at a concentration of 10^6^ cells/ml. PBMCs (200,000) with 2 μg/ml the concentration of a single peptide in the pool or 400 T cells/clone with 20,000 peptide-pulsed Epstein–Barr virus transformed B cells were used in standard human IFNγ ELISPOT assays as described elsewhere (15). In brief, assays were performed in 96-well MultiScreen filter plates (Merck Millipore, Watford, Hertfordshire, UK) coated with 10 μg/ml anti-IFN-γ (1-DIK, Mabtech, Nacka Strand, Sweden). Phytohemagglutinin (5 μg/ml, PHA, final concentration 1 μg/ml; Alere, Stockport, Cheshire, UK) was used as a positive control. Plates were incubated for 16 h at 37°C and 5% CO_2_. Spot enumeration was performed with an AID ELISPOT reader system (Autoimmun Diagnostika GmbH, Ebinger Strasse, Straßberg, Germany). To quantify antigen-specific responses, mean spots of the control wells were subtracted from the positive wells, and the results are expressed as SFU/10^6^ PBMCs. Responses were considered positive if results were at least three times the mean of the quadruplicate negative control wells and >25 SFU/10^6^ PBMCs. If negative control wells had >30 SFU/10^6^ PBMCs or positive control wells (PHA stimulation) were negative, the results were excluded from further analysis.

### Depletion of CD8^+^ T cells

CD8^+^ T and CD4^+^ T cells were depleted with M-450 Dynabeads (Invitrogen, Dynal, Oslo, Norway) according to manufacturers’ instructions. This method has been validated and widely used (15). Briefly, PBMCs from the same patient were divided and incubated with anti-CD8 or anti-CD4 mAbs conjugated to ferrous beads in 0.1% FCS PBS medium at 4°C for 30 min. The CD8^+^ and CD4^+^ T cells were removed using a magnet stand (Invitrogen, Dynal). The efficiency of depletion was assessed using a CyAn™ ADP flow cytometer (Dako, Ely, UK) and FlowJo software (Tree Star Inc., Ashland, OR, USA). The frequency of CD8^+^ T cells and CD4^+^ T cells was <1% after depletion.

### Tetramer staining and multicolor flow cytometry

Cryopreserved PBMCs were thawed as described above. A total of 1 × 10^6^ live PBMCs were labeled with tetramer-PE:HLA-A*0201 complexed with M1_58–66_ peptide GILGFVFTL, produced in-house using standard methods (20), and incubated for 15 min at 37°C. Cells were then incubated with CD8-PerCP and CD4-Pacific Blue (eBiosciences, Hatfield, UK), as well as a panel of antibodies for cell activation and differentiation markers: CD28-FITC, HLA-DR-APC, CD38-PE-Cy7, and CD27-APC-H7. Cells allocated to the intracellular panels were permeabilized with Perm/fix (BD, Oxford, UK) for 15 min and washed twice with 1× perm/washing buffer (BD). Cells were then labeled with Perforin-FITC (D48, Genprobe, Manchester, UK) or GranzymeA-FITC and GranzymeB-PB (Biolegend, London, UK). Cells were subsequently washed twice with 1× perm/washing buffer and fixed in BD cellfix (BD). All antibodies were from Becton Dickinson (BD, Oxford, UK) unless otherwise stated. Cell events were acquired on a nine-color CyAn Cytometer (Dako, Ely, UK), and data files were analyzed using FlowJo software. Data were analyzed using a forward side scatter gate followed by CD8 gating, then tetramer gating within the CD8^+^ population. These cells were then analyzed for percentage expression of a particular marker using unstained and CD8^+^tet^−^ populations to determine where to place the gates. Single-color samples were run for compensation, and fluorescence minus one (FMO) control samples were also applied to determine positive and negative populations, as well as channel spillover.

### T cell clones and EBV-transformed B cell line

Cytotoxic T cell (CTL) clones specific for peptide H1 HA-56 were generated by limiting dilution from the PBMCs of study subject ID24 and maintained as described by Dong et al. (21). An autologous EBV-transformed B cell line was also generated from this subject.

### Intracellular staining and flow cytometry

The following directly conjugated monoclonal antibodies were obtained from BD Biosciences (BD, Oxford, UK): IFN-γ (FITC), TNF-α (APC), CD107a (PE), CD3 (APC-H7), and CD8 (PE-Cy7). Antigen-specific CD8^+^ T cell clones were stimulated with peptide-pulsed autologous B cells in the presence of anti-CD107a for 1 h and incubated with 0.7 μg/ml monensin (BD Biosciences) and 10 μg/ml Brefeldin A (BD Biosciences) for an additional 5 h at 37°C. Negative controls included un-stimulated cells. CD8^+^ T cell populations producing cytokines were fixed and stained as described above and detected by flow cytometry.

### Statistical analysis

All statistical analyses were performed using Prism 6 (GraphPad Software). *p-*values were calculated using the Wilcoxon matched-pairs signed rank test or the Mann–Whitney test. *p* < 0.05 was regarded as statistically significant.

## Results

### Study subjects and specimens

**Figure 1:**
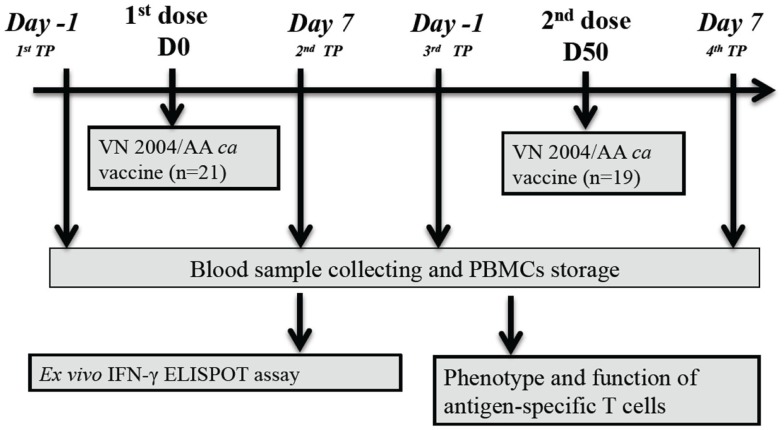
**Flow diagram of the clinical trial study design**.

As described by Karron et al. (4), 21 healthy volunteers were enrolled in this phase I clinical trial and received the H5N1 VN 2004 ca vaccine intranasally. With the exception of 2 study subjects (ID31 and ID41), the remaining 19 study subjects received a second dose of vaccine approximately 50 days later. As shown in Figure 1, blood samples were taken from each study subject at four time points (TP): pre-vaccination (first TP), 7 days after the first dose (second TP), 1 day prior to the second dose (third TP), and 7 days after the second dose (fourth TP). Stored frozen PBMCs were used for this study. Infection with vaccine virus occurred in 12 study subjects.

### H5N1 pLAIV vaccine boosts influenza-specific T cell responses

**Figure 2:**
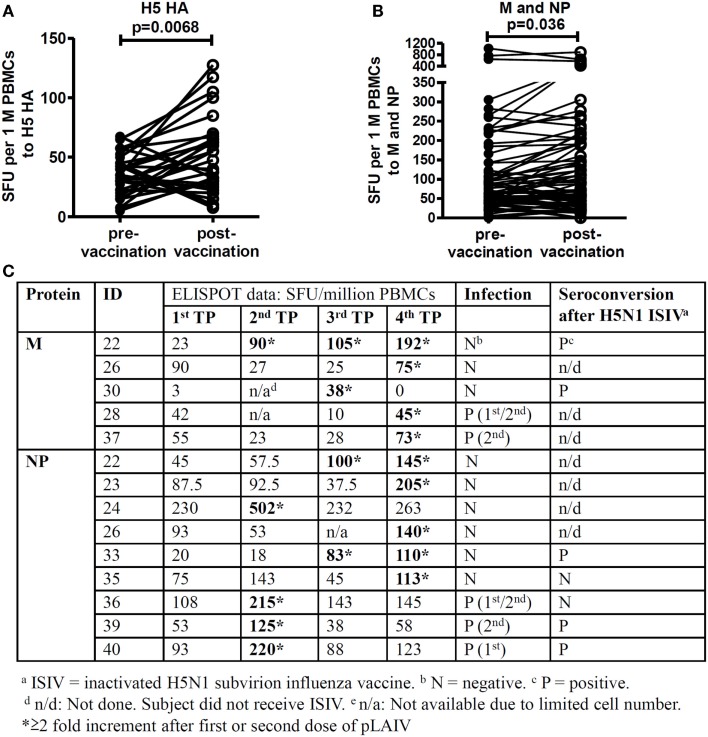
**Elevated T cell responses after each vaccination**. T cell responses at all four time points (TP) were screened by *ex vivo* IFN-γ ELISPOT using overlapping peptides fromH1 HA, H3 HA, H5 HA, and H5N1 VN 2004 Matrix proteins (M1 and M2) and Nucleoprotein. *n* = 2 replicates. **(A)** Comparison of T cell responses targeting H5 HA peptides pre- and post- first and second vaccination (*n* = 21). **(B)** Comparison of T cell responses targeting internal proteins M (*n* = 20) and NP (*n* = 19) pre- and post- first and second vaccination. *p-*values were calculated using the Wilcoxon matched-pairs signed rank test. **(C)** Study subjects who showed >2-fold elevated T cell responses to M and NP peptides after vaccination.

Significantly elevated T cell responses were observed to H5 HA (*p* = 0.0068, Figure 2A) after first and second dose of vaccine; elevated T cell responses to M and NP proteins were also observed (Figure 2B, *p* = 0.036) (Figure 2B). We found that 12 of 21 study subjects showed elevated T cell responses to the highly conserved M and NP proteins after the first and/or second dose of pLAIV, regardless of whether they had confirmed vaccine virus infection (Figure 2C). These responses did not correlate with the antibody responses following ISIV boost administered in a follow-up study (18).

### The H5N1 pLAIV is able to stimulate cross-reactive T cell responses with an effector phenotype, specific to internal viral proteins

**Figure 3:**
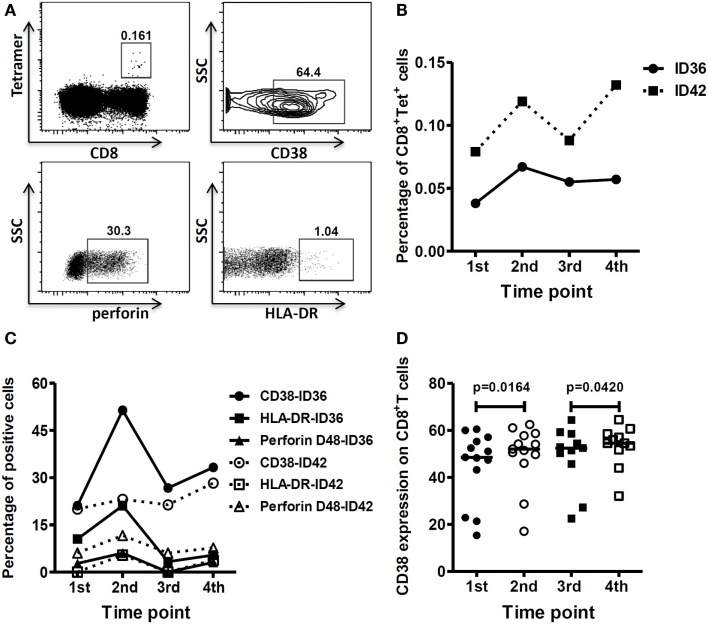
HLA-A*0201 M1_58–66_-specific T cells were boosted and showed better effector function after each vaccine dose. **(A)** Gating strategy. Gating of CD8^+^ tetramer^+^ cells, CD8^+^ CD38^+^ cells, CD8^+^ perforin^+^ cells, and CD8^+^ HLA-DR^+^ cells. The gating of CD38^+^, Perforin^+^, and the HLA-DR^+^ population on antigen-specific T cells is same as the gating on CD8^+^ T cells. The percentage of tetramer^+^ cells shown is within CD8^+^ T cells. **(B)** Frequency of HLA-A*0201 M1_58–66_ tetramer positive cells before and after each vaccine dose. **(C)** Expression of activation markers (CD38 and HLA-DR) and cytolytic marker (Perforin D48) on HLA-A*0201 M1_58–66_ tetramer positive cells before and after each vaccine dose. Lines represent the percentage of tetramer positive cells with the noted markers. **(D)** Comparison of CD38 expression on CD8^+^ T cells before and after each vaccine dose in the vaccine virus-infected group (first vaccination: *n* = 13; second vaccination: *n* = 11). *p-*Values were calculated using the Wilcoxon matched-pairs signed rank test. For the scatter dot plots, the line represents the median value.

We next evaluated the phenotype of antigen-specific CD8^+^ T cells by staining PBMCs with an MHC class I tetramer specific to an HLA-A0201-restricted M1 protein (58–66) epitope and a panel of antibodies specific for cell activation and cytotoxicity markers. Figure 3A displays the gating strategy used in flow cytometry. T cells from two study subjects (ID36 and ID42) who were infected with the vaccine virus were positively stained with this tetramer. Figure 3B clearly demonstrates that the proportion of CD8^+^ tetramer^+^ T cells increased after vaccination. In study subject ID36, the CD8^+^ tetramer^+^ T cells expanded from 0.038 to 0.067% after the first dose of vaccine. Although the size of the antigen-specific T cell population shrank slightly thereafter, from 0.067% 7 days after the first dose of vaccine to 0.057% 7 days following the second dose of vaccine, it was still greater than the baseline level. In study subject ID42, the CD8^+^ tetramer^+^ T cells were boosted after each dose of vaccine, with an approximately 0.05% increase post-vaccination. The number of antigen-specific T cells increased, and there was an enhancement in expression levels of cell activation and cytotoxicity molecules, such as CD38, HLA-DR, and perforin, on the T cells (Figure 3C), indicating that the H5N1 pLAIV could boost CD8^+^ T cells specific to internal viral proteins with effector functions. Moreover, we also observed stronger systematic activation of CD8^+^ T cells from study subjects who were infected with the vaccine virus. As shown in Figure 3D, the expression level of CD38 on the surface of CD8^+^ T cells was higher after each dose of vaccine compared to pre-vaccination levels.

### Elevated HA-specific T cell responses to seasonal influenza viruses with low cross-reactivity to H5N1 HA peptides

**Figure 4:**
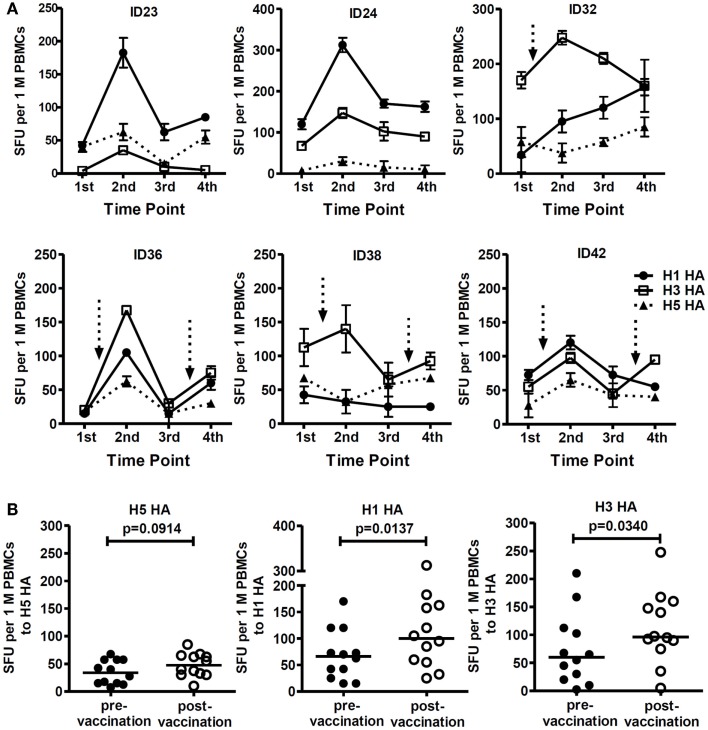
**T cell responses to seasonal influenza HA peptides were elevated after the first and second dose of the pLAIV**. **(A)** T cell responses to H1 HA, H3 HA, and H5 HA peptides at all four time-points in six study subjects. *n* = 2 replicates; Arrows represent documented vaccine virus infection. **(B)** Comparison of T cell responses targeting H5 HA, H1 HA, and H3 HA pre- and post- first and second vaccination, *n* = 6. *p*-Values were calculated using the Wilcoxon matched-pairs signed rank test. For the scatter dot plots, the line represents the median value.

We also observed elevated T cell-specific responses to HA proteins of seasonal influenza viruses (H1 and H3) in 6 out of 21 subjects following receipt of the H5 pLAIV vaccine (Figure 4A). The enhanced responses to seasonal influenza virus HAs, particularly H1 HA, were higher than the responses to the H5 HA protein (Figure 4B), indicating that the H5N1 pLAIV preferentially boosted T cell responses to seasonal influenza HA proteins rather than H5N1 HA in some individuals.

**Figure 5:**
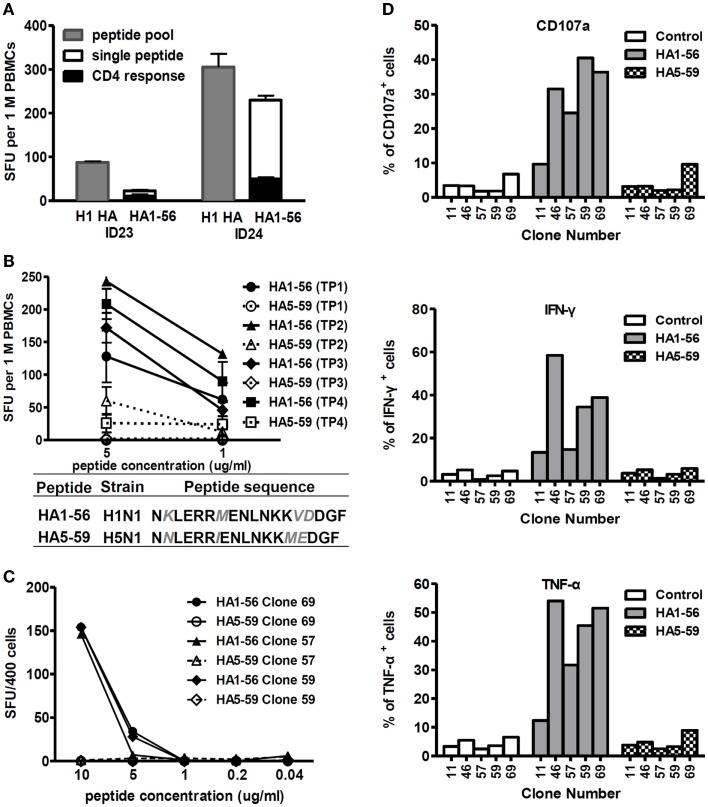
**T cell responses to seasonal influenza HA proteins showed a low level of cross-reactivity to H5 HA**. **(A)** T cell responses to peptide pools, single peptide, and responses to single peptide elicited by CD4^+^ T cells from study subjects ID23 at time point 2 and ID24 at time point 4. *n* = 3 replicates. **(B)** Cross-recognition of peptides HA1-56 and HA5-59 by T cells from study subject ID24 at all four time points. *n* = 3 replicates. Amino acids differing between HA1-56 and HA5-59 are shown in gray italics. **(C,D)** Cross-recognition of peptides HA1-56 and HA5-59 by T cell clones generated from study subject ID24. The cross-reactivity of T cell clones was assessed by IFN-γ ELISPOT using titrated peptides **(C)**, degranulation (CD107a expression), and intracellular TNFα and IFN-γ production **(D)**.

As illustrated in Figure 5A, both study subjects ID23 and ID24 displayed responses to peptide HA1-56 from the H1 HA protein, and the responses were mainly elicited by CD8^+^ T cells. However, our *ex vivo* ELISPOT data showed that the T cells did not recognize the corresponding peptide from the H5 HA, known as HA5-59 (Figure 5B). Subsequently, we generated CD8^+^ T cell clones specific to the H1 HA1-56 peptide from study subject ID24 and tested their cross-reactivity to the H5 HA5-59 peptide. As shown in Figures 5C,D and Figure S1 in Supplementary Material, all three T cell clones were capable of degranulation and producing IFN-γ and TNF-α when stimulated by peptide HA1-56. However, they did not show any responses to peptide HA5-59, even at a high peptide concentration. These data suggest that T cell responses to seasonal influenza HA proteins had low-level cross-reactivity to the H5 HA, an example of original antigenic sin (OAS) for T cells.

### High pre-existing cross-reactive responses to internal influenza virus proteins do not restrict infectivity of the vaccine virus

**Figure 6:**
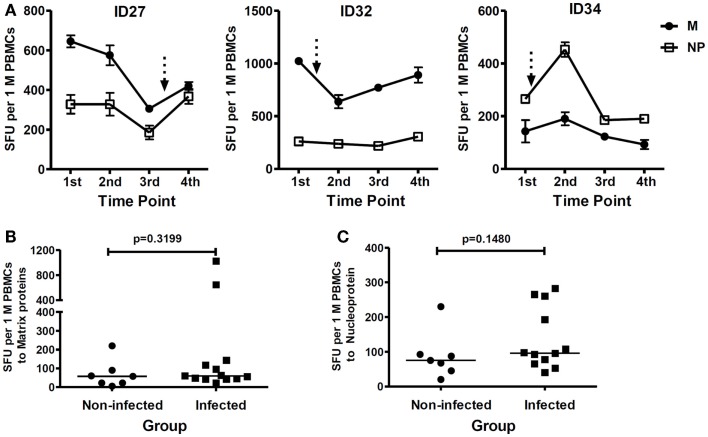
**T cell responses targeting H5N1 internal viral proteins**. **(A)** Examples of the study subjects from the vaccine virus-infected group (ID27, ID32, and ID34) who showed high pre-existing cross-reactive T cell responses. *n* = 2 replicates. Arrows represent documented vaccine virus infection. **(B)** Comparison of pre-existing cross-reactive T cell responses targeting the viral M proteins between the vaccine virus-infected (*n* = 13) and un-infected (*n* = 7) study subjects. **(C)** Comparison of pre-existing cross-reactive T cell responses targeting NP protein between the vaccine virus-infected (*n* = 12) and un-infected (*n* = 7) study subjects. Study subject *ID29* was excluded because of limited cell numbers*. p-*values were calculated using the Mann–Whitney test. For the scatter dot plots **(B,C)**, the line represents the median value.

On screening for T cell responses to internal influenza virus proteins, we observed strong cross-reactive responses to internal proteins of the H5N1 virus, especially M and NP, in some infected study subjects prior to immunization. For example, as illustrated in Figure 6A, study subjects ID27 and ID32 who were infected with the H5N1 pLAIV vaccine, showed strong T cell responses to the M protein before vaccination, with a level of IFN-γ production >600 SFU/10^6^ PBMCs. Among the study subjects infected with the pLAIV, pre-existing T cell responses targeting the NP protein were also detected in ID27, ID32, and ID34, with a magnitude >250 SFU/10^6^ PBMCs. There was no significant difference in the pre-existing T cell responses targeting internal viral proteins M and NP between the pLAIV-infected and un-infected groups (Figures 6B,C, and data not shown). These data indicate that high pre-existing cross-reactive responses to internal influenza viral proteins are unlikely to have played a role in restricting the infectivity of the vaccine virus.

## Discussion

Vaccination with the H5N1 pLAIV stimulated modest influenza-specific T cell responses in most vaccine recipients. The responses were in both CD4^+^ and CD8^+^ T cells and the T cells showed evidence of cytolytic function. There was no relationship between the T cell responses and other evidence that of pLAIV vaccine infection, either by PCR-detected shedding or a fourfold rise antibody titer. Although the H5N1 pLAIV was highly restricted in replication and was poorly immunogenic in the phase I clinical trial, we recently showed that the H5N1 pLAIV induced long-term immune memory (18). We detected a high titer, rapid antibody response in most of the study subjects following the administration of a single dose of an H5N1 inactivated subunit influenza vaccine (ISIV) almost 5 years after the initial H5N1 pLAIV (18). Interestingly, pLAIV priming of these antibody responses occurred even in the absence of significant vaccine virus shedding and immunogenicity measured by traditional end points in the initial phase I clinical trials of the H5N1 pLAIV (18). In the current study, indications of antigen exposure by significantly elevated T cell responses were observed after the first and/or second dose of pLAIV in most volunteers; these responses did not correlate either positively or negatively with the antibody responses following ISIV boost. In a separate study in Vietnam, we have detected H5N1-specific T cell responses in a village cohort with H5N1 virus exposure, regardless of the detection of antibodies (22). Thus, it is likely that exposure to infectious influenza virus can sometimes stimulate CD8^+^ T cell responses without inducing antibody responses or infecting sufficient cells in the respiratory tract to be detectable by PCR or virus culture. Detection of influenza virus-specific T cell responses may serve as an additional marker for subclinical H5N1 virus infection in humans.

T cell immune responses were detected targeting internal viral proteins, which are highly conserved between different influenza virus strains. These highly cross-reactive T cells are likely to confer broader or potentially “universal” protection against a wide range of influenza viruses (19). T cell responses, especially cross-reactive T cell responses, correlate with protection in several studies, including our own (14, 15). Although very low antibody responses were detected in pLAIV study subjects (9), our results showed elevated T cell responses in >60% of study subjects in response to at least one influenza virus internal protein (mostly to M and NP protein, >2-fold increase) after the first and/or second dose of vaccine. However, some increased responses were seen 50 days after the first vaccination but not at 7 days, suggesting that further optimization of the timing of the T cell assays after LAIV administration might be needed. Taken together, the advantages and potential of evaluating T cell responses against internal viral proteins, especially M and NP, along with neutralization and HAI antibody responses might be considered for future evaluation of vaccine immunogenicity.

Elevated HA-specific T cell responses to seasonal H1 and H3 influenza viruses, with low cross-reactivity to H5N1 HA peptides were detected in study subjects after the first or second dose of H5N1 pLAIV, suggesting an “original antigenic sin” phenomenon. Original antigenic sin in T cells in humans was first described for dengue viruses by Mongkolsapaya et al. (23) and implies that the response to a secondary infection by a dengue virus is dominated by the proliferation of cross-reacting memory T cells induced by primary infection with a different viral strain, which is of lower affinity for the secondary viral antigen. However, whether this will be to the benefit or the detriment of the host remains unanswered.

Finally, the presence of high level pre-immunization T cell responses in three volunteers did not prevent boosting of T cell responses. Therefore, this does not appear to be the reason why the pLAIV did not infect all the study subjects. However, as discussed above, the vaccine boosted T cell responses in the absence of detectable virus shedding or a rise in antibody titer. It is likely therefore that low-level infection by the attenuated pLAIV rather variably stimulates both T and B cell responses.

## Conflict of Interest Statement

The authors declare that the research was conducted in the absence of any commercial or financial relationships that could be construed as a potential conflict of interest.

## Supplementary Material

The Supplementary Material for this article can be found online at http://journal.frontiersin.org/article/10.3389/fimmu.2015.00287/abstract
