# Supplementary figures and images for: Boosted Influenza-Specific T Cell Responses after H5N1 Pandemic Live Attenuated Influenza Virus Vaccination
Source: Front Immunol. 2015 Jun 2;6:287. doi: 10.3389/fimmu.2015.00287 (PMC4451682; doi:10.3389/fimmu.2015.00287)

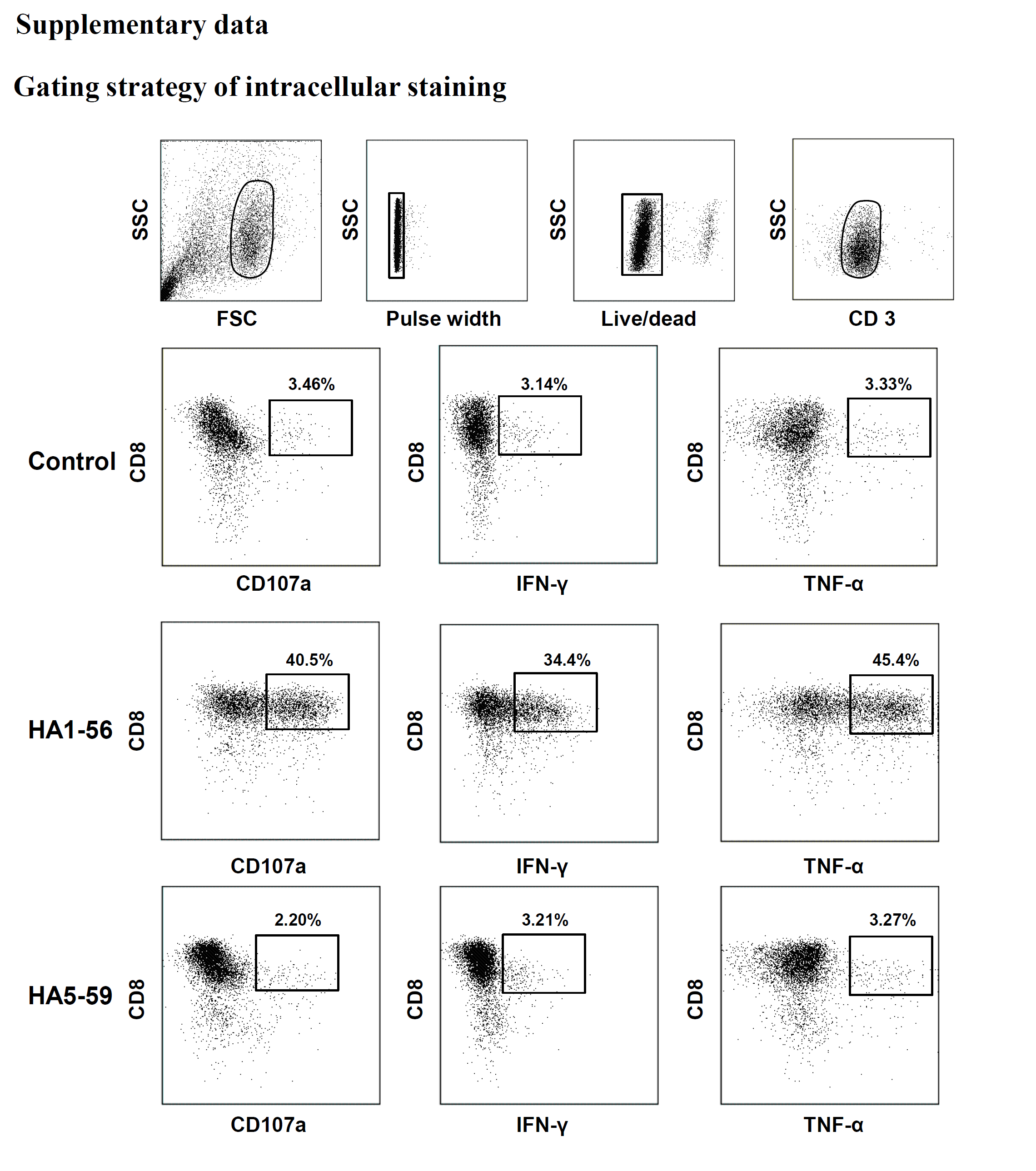

Supplement: Supplementary file 1 [file image_1.tif]
